# Effect of chrono-moxibustion and its influence on circadian rhythm for rheumatoid arthritis: A study protocol for a randomized controlled trial

**DOI:** 10.1097/MD.0000000000030701

**Published:** 2022-09-23

**Authors:** Mingfang Yu, Shenqiao Yang, Bailu Chen, Lu Gan, Xinling He, Aiyang Wang, Xiao Wu

**Affiliations:** a The Affiliated Traditional Chinese Medicine Hospital of Southwest Medical University, Luzhou, Sichuan, China; b Luzhou Hospital of Traditional Chinese Medicine, Luzhou, Sichuan, China; c Chengdu University of Traditional Chinese Medicine, Chengdu, Sichuan, China.

**Keywords:** chrono-moxibustion, circadian rhythm, randomized controlled trial, rheumatoid arthritis (RA), study protocol

## Abstract

Rheumatoid arthritis (RA) is a chronic disease which is characterized by a circadian variation of key clinical symptoms and findings, with prominent joint swelling, stiffness and pain occurring in the early morning and light clinical symptoms during the day. Chrono-moxibustion is carried out at different time, which could result in dissimilar therapeutic effects. However, its efficacy has seldom been systematically demonstrated and few studies have reported that Chrono-moxibustion may regulate the circadian rhythm of RA. We therefore designed a randomized trial to explore the effective difference of Chrono-moxibustion in RA treatment, as well as to study its influence on circadian rhythm of RA patients.

**Methods:**

This study is a randomized controlled trial involving 120 participants, and a total of 90 eligible RA patients will be randomly allocated to three groups in a 1:1:1 ratio as moxibustion at 7 to 9 am, moxibustion at 5 to 7 pm, and waiting list group, meanwhile, 30 healthy people will be divided into the control group. Patients in moxibustion groups will be treated for 30 minutes per session, 3 times a week, lasting 6 weeks. All of RA patients will be evaluated with questionnaires and laboratory tests before treatment, as well as 3 weeks, 6 weeks, and 3 months after treatment. One way analysis of variance (ANOVA) with multiple comparisons will be applied to identify differences more than two groups. Halberg cosiner software will be used to analysis the circadian rhythm.

**Results:**

The results of this study will be published in a peer-reviewed journal.

**Conclusion:**

This study will provide evidence-based evidence for the effective difference of Chrono-moxibustion in RA treatment and its influence on circadian rhythm of RA patients.

## 1. Introduction

Rheumatoid arthritis (RA) is a chronic autoimmune disease characterized by progressive and persistent synovitis. The main feature of its clinical manifestations possess circadian variation which shows light symptoms during the day and heavy symptoms at night.^[1]^ On the other hand, Previous findings suggested that the rhythm variations of clinical feature were attributed to the circadian oscillation of key inflammatory cytokines in RA. The Global Burden of Disease Study (2017)^[2]^ presented that the age-standardized prevalence and incidence rates of RA were increasing. RA affected 0.5% to 1.0% of the global population, seriously reduced life quality and damaged the physical and psychological health.^[3]^ Because of its complex etiology and pathology, protracted and difficult to recover, it has become one of the major difficult diseases concerned by all the medical field.

Currently, there are four main strategies of the approved RA treatment, including non-pharmacological treatments, non-steroidal antiinflammatory drugs (NSAIDs), disease-modifying antirheumatic drugs (DMARDs), and Glucocorticoids.^[4]^ While, there are also some recommended non-pharmacological treatments, such as physical therapy, functional exercise, patient counseling and complementary and alternative medicine.

NSAIDs can effectively relieve pain, swelling and improve joint function, while Glucocorticoids has rapid disease-modifying effects and DMARDs can ease symptoms and restrain the joint destruction clinically.^[5]^ Nevertheless, all of these currently available drugs showed severe side-effects and high cost.^[6]^ Hence, many RA patients are seeking complementary and alternative medicine such as acupuncture and moxibustion.

As a safe and effective therapy of traditional Chinese medicine (TCM), moxibustion has been widely adopted in the RA treatment for many years.^[7,8]^ Previous studies certified that moxibustion, which can significantly alleviate pain, swelling and stiffness, are effective in treating RA.^[9–11]^

In addition, chronotherapy has been developed in order to better fit circadian variations of clinical feature, improve efficacy and minimize adverse events (AEs). For instance, the modified-release prednisone was administered in the evening at 10.00 pm, which seemed more effective in ameliorating morning stiffness in RA patients.^[12]^

Acupuncture and moxibustion were performed according to time, which has been used clinically for thousands of years in China. As a consequence, the achievements of chronotherapy in Western medicine have prompted us to combine the RA pathological mechanism with Chrono-moxibustion which was conducted at different time. Our pilot study also suggested that Chrono-moxibustion can engender dissimilar therapeutic effects.^[13]^ Nevertheless, there is a lack of rigorous clinical randomized controlled trial to clarify dissimilar therapeutic effect of Chrono-moxibustion on RA. Besides, the clinical study of Chrono-moxibustion regulating the circadian rhythm of RA is relatively unavailable. Consequently, we will study the effectiveness of Chrono-moxibustion and explore its influence on circadian rhythm of RA patients.

## 2. Methods

### 2.1. Study design

The study aims to assess the effectiveness of moxibustion for RA, as well as to determine which time is more optimal in clinical practice. In addition, it will explore the influence of Chrono-moxibustion on circadian rhythm of RA patients. According to the validity of Chrono-moxibustion may adjust circadian rhythm of RA patients, we hypothesized there will be diverse effect by Chrono-moxibustion based on the theory of Chronobiology, moreover, it can also modulate the pathological rhythm of RA.

### 2.2. Study setting

**Figure 1.**
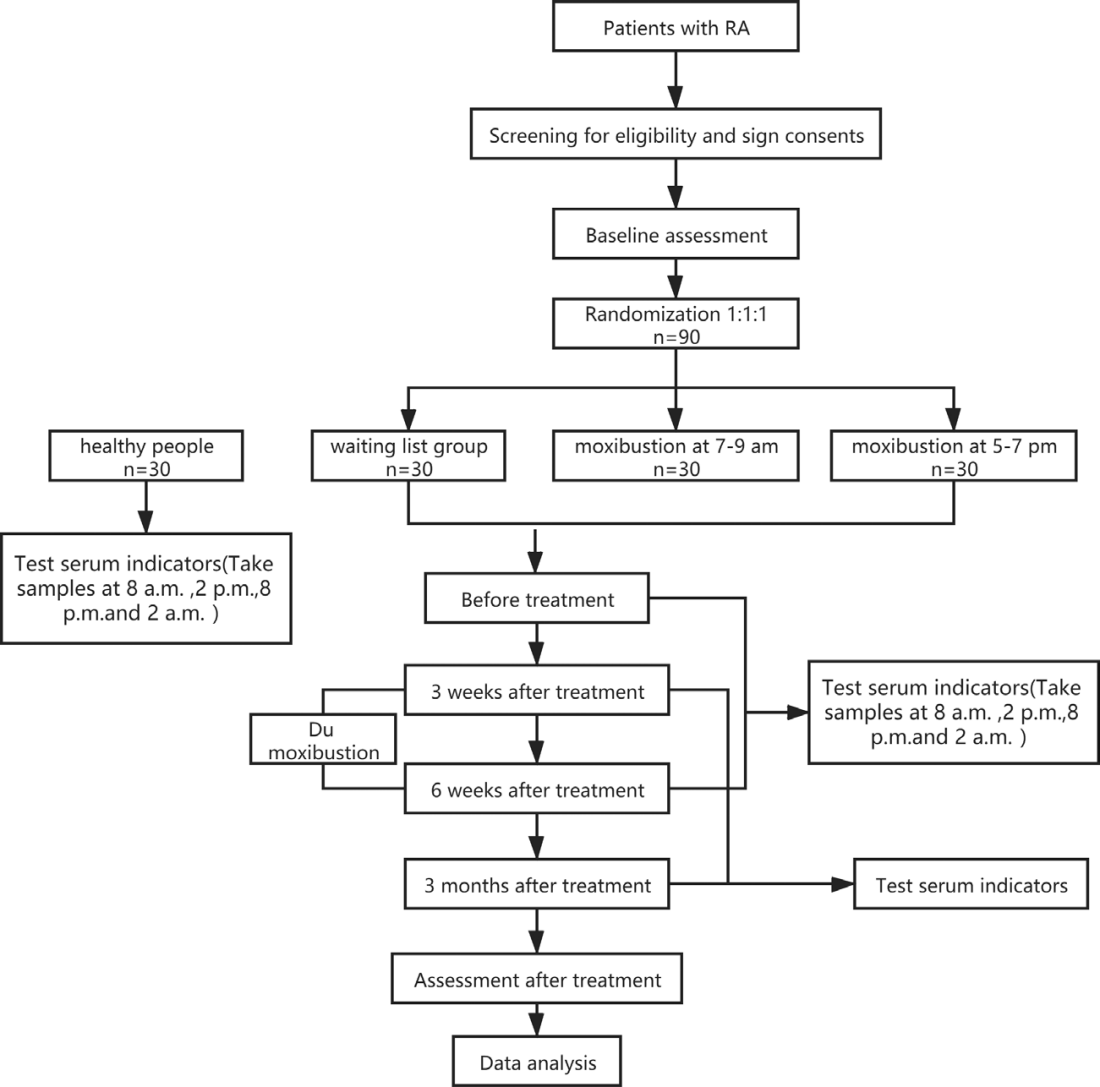
Study flowchart. Serum indicators include ESR, CRP, RF, IL-1, IL-6, TNF-, IL-18, MMP-3. ESR = erythrocyte sedimentation rate, CRP = serum C-reactive protein, RA = rheumatoid arthritis.

A total of 90 RA patients will be divided randomly into 3 groups via a completely randomized digital table as moxibustion at 7 to 9 am (group A), moxibustion at 5 to 7 pm (group B) and waiting list group (group C) by a ratio of 1:1:1, meanwhile, 30 healthy people will be divided into control group (group D). This RCT will be conducted in multicenter with the assessor and statistician blinded to treatment allocation, and will be carried out at 4 hospitals in China: The Affiliated TCM Hospital of Southwest Medical University, the Luzhou Hospital of Traditional Chinese Medicine, Hejiang Hospital of Traditional Chinese Medicine and Rongchang District Hospital of Traditional Chinese Medicine. The specific process of participant inclusion is illustrated in Figure 1.

### 2.3. Patients recruitment

There will be 3 prime strategies to recruit RA patients. The first strategy is to recruit participants from the outpatient and inpatient. Second, printed recruitment posters will be distributed in public clinics and nearby communities to enroll potential eligible study subjects. Third, we will post advertisements, through the Internet, social software, and so on, to briefly introduce our study, so as to attract possible patients who are willing to participate. There are two researchers concerning recruitment and registration of participants who meet the inclusion criteria.

### 2.4. Randomization and allocation concealment

Eligible patients will be randomly assigned to 3 groups in a 1:1:1 ratio. The computer-generated random number list will be concealed in sequentially numbered, opaque, and sealed envelopes which kept by an independent custodian to ensure allocation concealment. The patients’ screening sequence numbers will be printed on the outside while the group names will be printed inside the envelope. The practitioner will firstly verify each patient meet the eligibility criteria and sign the written informed consent form, and then open the sealed envelope which containing an allocation sequence number for each patient. If any error or disclosure regarding randomization occurs, a new randomization sequence will be generated starting from the problematic serial number and applied to subsequent patients from then on.

### 2.5. Blinding

Taking into account that the significant difference of treatment and the specificity of moxibustion, patients will understand their group allocation, so will the operators, meaning that double blinding the study is unfeasible. Consequently, we will strictly carry out random allocation in order to ensure the integrity of this research, meanwhile, the efficacy assessors and outcome statisticians should be blinded to avoid the bias resulting from subjective impression.

### 2.6. Inclusion criteria

Patients meeting all of the following criteria will be enrolled in the study:

1. Patients meeting the diagnostic criteria for RA according to the 2010 criteria of the American College of Rheumatology (ACR)/European League Against Rheumatism (EULAR) Collaborative Initiative,^[14]^ which means classification as “Diagnosis of RA” is based on the presence of synovitis in at least 1 joint, and the total score of 6 or higher according to the individual scores in 4 fields: number and location of affected joints (score range 0–5), serological abnormality (score range 0–3), synovitis duration (score range 0–1), acute phase related reactants (score range 0–1).
2. Chief signs and symptoms are joint swelling, tenderness, and stiffness.
3. Female or male aged from 18 to 65.
4. Voluntarily participating in this study with a written informed consent form signed by the participants themselves.
5. Participants can understand the scales, that is, which are used to measure trial outcomes.

### 2.7. Exclusion criteria

Subjects will be excluded if they meet one of the following criteria:

1. Overlap other rheumatic diseases such as systemic lupus erythematosus, Sjogren syndrome, severe knee osteoarthritis, etc.
2. Severe extra articular manifestations, such as persistent high fever, interstitial pneumonia, renal amyloidosis, constrictive pericarditis, central nervous system vasculitis, which need to be treated with glucocorticoid.
3. At present, patients who are participating in or have participated in other clinical studies on the treatment of RA within 1 month before this study; acupuncture or moxibustion treatment for the disease during the past 1 month.
4. Severe primary disease or infectious diseases including kidney, heart or liver disease, hematopoietic system, endocrine system disease, or pregnancy or breastfeeding.
5. A severe psychological disorder or psychiatric condition associated with dementia and severe neurosis and inability to communicate or take care of oneself.
6. With active gastrointestinal disease or peptic ulcer within 30 days before the study.
7. Any other condition that the investigator consider as likely to make the patient incapable to complete, comply, or unsuitable for the clinical trial.

### 2.8. Withdrawal or dropout criteria

1. The participant has an AE related to the research.
2. Those who can’t be judged the curative effect, or whose data are incomplete to affect the measurement of curative effect or safety.
3. Although they met the inclusion criteria and were included, they did not receive any treatment related to this trial.
4. At the participant’s own request.

### 2.9. Procedure

Potential participants will be initially screened according to the inclusion and exclusion criteria by our recruitment team, and those who may meet the eligibility requirements will be subjected to RA related serological testing for secondary evaluation. Eligible participants who are willing to take part in this trial will be asked to sign a written informed consent form after completing the screening tests. Then, all of eligible patients will be randomized to group A, group B, or group C. After randomization, subjects enter a 1-week baseline period then they will complete the baseline questionnaire and blood sampling at 4 equidistant time points within 24 hours. Subsequently, they will receive a 6-week moxibustion in the light of their group allocation and then provide blood specimens at 4 equidistant time points within 24 hours. After 3 weeks and 3 months, blood sample will be also collected. All the RA subjects will be evaluated with study-related scales before treatment, as well as 3 weeks, 6 weeks and 3 months after treatment. Three months after the end of treatment, all participants will be followed up to assess the curative effect and safety (Table 1).

**Table 1:** The schedules for patient enrollment, interventions and assessments. SF-MPQ, short-form Mcgill pain questionnaire; DAS28, disease activity score of 28 joints.

Study schedule					
	Baseline	Treatment phase (Weeks 1–6)			Follow-up phase
Timepoint	Week –1	Week 0	Week 3	Week 6	Month 3
Patients					
Enrollment	√				
Informed consent	√				
Signed informed consent		√			
Medical history	√				
Physical examination	√				
Laboratory test	√				
Randomization		√			
Intervention					
Moxibustion at 7–9 am		√	√	√	
moxibustion at 5–7 pm		√	√	√	
Waiting list group					
Outcomes					
SF-MPQ	√		√	√	√
Laboratory indicators	√		√	√	√
Joints morning Stiffness score	√		√	√	√
Joints swelling index	√		√	√	√
Joints tenderness index	√		√	√	√
DAS28	√		√	√	√
Monitor					
Adverse events					√
Patient’s compliance					√
Reasons of drop-outs or withdrawals					√

DAS28 = disease activity score 28, SF-MPQ = Short-Form McGill Pain Questionnaire.

### 2.10. Interventions

**Figure 2.**
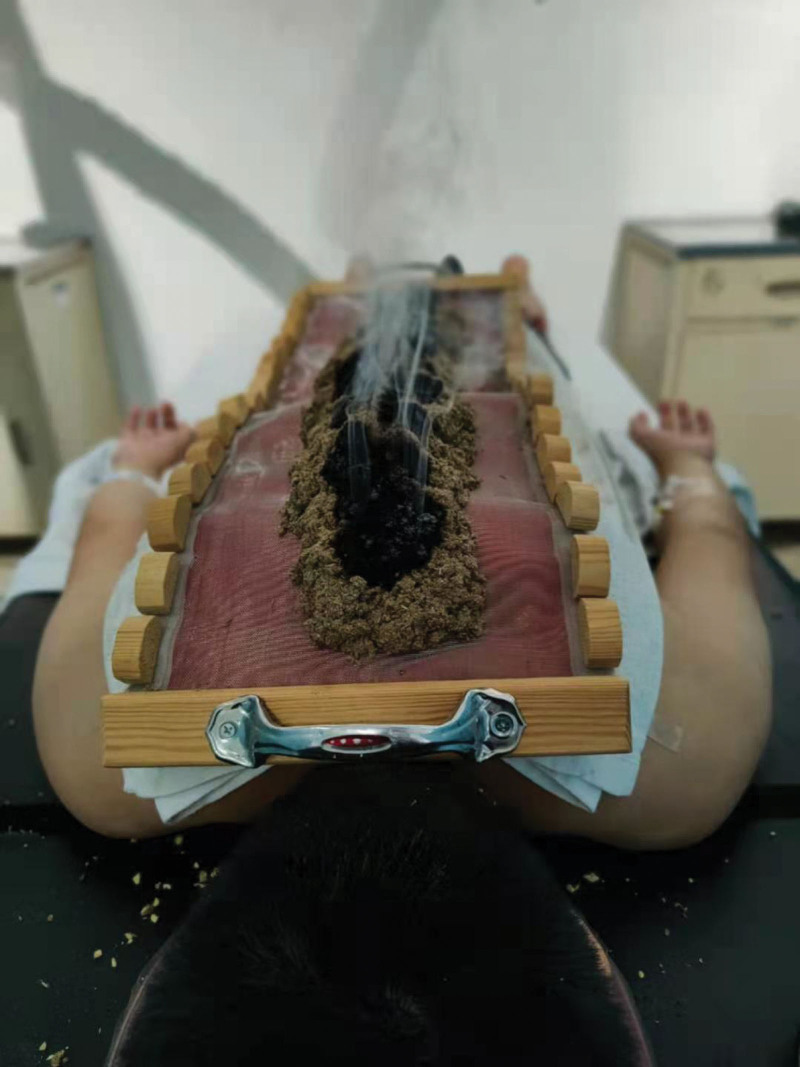
Du moxibustion.

Moxibustion staff members who have more than one year experience in Du moxibustion will be called for this trial and retrained for standard operation before the trial. The moxibustion acupoints are identified in accordance with the method of acupoint location issued by the World Health Organization (WHO).^[15]^ Du moxibustion treatment will be carried out from DU14 (Dazhui) to DU1 (Changqiang). The patient will be in prone position with bare back and be covered with a strip of gauze to prevent scalding. Large and long moxibustion box (60 cm long) will be placed on the back to cover the acupoints. Place the moxa velvet evenly in the moxibustion box (about 3 cm in height and 2 cm in width), ignite the moxa velvet from the top, and let it burn from top to bottom. Remove the ash of moxa after it is burned out (Fig. 2). The total time of moxibustion each time is about 30 minutes. After the treatment, the moxibustion box will be removed, with the degree of local skin flush and no blister. Moxibustion treatment will be conducted 3 times a week for 6 weeks. The patients will be followed up for 3 months. Patients in group A and B will be treated with Du moxibustion at 7 to 9 am and 5 to 7 pm, respectively. The group C will not receive any adjunctive treatment during the trial period, and then accept moxibustion treatment after the investigation. During the trial, the patients in each group will maintain the routine drug treatment.

### 2.11. Outcomes

All of the outcomes will be assessed for 4 times before the treatment, as well as 3 weeks, 6 weeks, and 3 months after treatment (Table 1).

#### 2.11.1. Primary outcome.

##### 2.11.1.1. Short-Form McGill Pain Questionnaire (SF-MPQ).

The SF-MPQ is a multidimensional measure of perceived pain in adults with chronic pain, including pain due to rheumatic diseases. It includes 3 items: the Pain Rating Index, Present Pain Intensity, and Visual Analog Scale (VAS) for average pain. The Pain Rating Index is comprised of 15 words (11 sensory and 4 affective), and each selected word is scored from 0 (none) to 3 (severe). The total Pain Rating Index score is obtained by summing the item scores (range 0–45). Scores on the Present Pain Intensity range from 0 to 5 and on the VAS from 0 to 10. A higher score on the SF-MPQ indicates worse pain.

#### 2.11.2. Secondary outcome measurement.

##### 2.11.2.1. Laboratory indicators.

In this study, all subjects will be measured for ESR, CRP, RF, IL-1β, IL-6, TNF-α, IL-18, MMP-3 in serum. ESR, CRP, and RF will be detected by automatic biochemical analyzer, while IL-1β, IL-6, TNF-α, IL-18, and MMP-3 via ELISA. Group D will be tested only one time during the study while RA patients will be tested before the treatment and 6 weeks after the treatment by a way that four equidistant time points within 24 hours for blood sampling. Except for this, RA patients will be examined after a 3 -week and 3- month intervention.

##### 2.11.2.2. Joints morning stiffness score.

It refers to the time required for the patient to start activities after waking up to the obvious relief of morning stiffness symptoms, calculated in minutes. Score calculation method: no morning stiffness, 0 point; morning stiffness time <1 hour, 2 points; 1 hour ≤morning stiffness time <2 hours, 4 points; Morning stiffness time >2 hours, 6 points. The higher the score, the more serious of the disease.

##### 2.11.2.3. Joints swelling index.

Joints swelling index is evaluated by observing 28 joints of the whole body to judge the degree of swelling. Grade the swelling of the joints: no swelling, 0 point; swelling lower than the nearby bone markers, 1 points; swelling as tall as the bone markers, 2 points; swelling higher than the nearby bone markers, 3 points. The joint swelling index, expressed as a sum, where each joint is graded for swelling on a scale of 0 none, 1 mild, 2 moderate, and 3 severe. The higher the score, the more serious the condition.

##### 2.11.2.4. Joints tenderness index.

Joints tenderness index is to judge whether there is tenderness and record the number of tenderness of 28 joints. It is graded as follows: no tenderness, 0 point; tenderness with flinch, 2 points; tenderness with flinch and avoidance, 3 points. The higher score, the more severe pain and dysfunction.

##### 2.11.2.5. Disease activity score of 28 joints (DAS28).^[16]^

DAS 28 is one of the most commonly and widely method to evaluate RA disease activity in clinic. It is comprised of tenderness and swelling of 28 joints such as shoulder, elbow, knee, wrist, MCPs, and PIPs. It is calculated by swollen joint count (SJC), tender joint count (TJC), a marker of inflammation like CRP or ESR, visual analog scale (VAS), general health (GH). DAS28 scores were categorized as low (<3.2), moderate (<5.1), or high (≥5.1). The higher the score, the higher the activity of the RA disease.

### 2.12. Sample size calculation

The sample size has been estimated based on the results of a preceding study that had shown that moxibustion can ease RA patients.^[10]^ Results from a previous study showed that the mean changes in the Disease activity score in 28 joints (DAS 28) were 4.17 ± 0.94 (moxibustion group) and 4.86 ± 1.40 (control group). PASS software was used to calculate the sample size. The confidence of the trial sample size was 90% and the significance level was 0.05. Results showed that clinically significant differences would be detected using a minimum sample size of 26 individuals in each group. The maximum permissible withdrawal rate is 15% and 120 participants (30 per group) will be recruited.

### 2.13. Statistical analysis

To eliminate artificial error, two statisticians blinded to the whole trail process will independently perform the statistical analysis by using SPSS statistics software (version 21) and Halberg cosiner software.

Baseline data including age, sex, disease duration, SF-MPQ score, joints morning stiffness score, joints swelling index, joints tenderness index, DAS28 score and laboratory indicators results before interventions will be analyzed with different approaches. All the data will be presented as mean ± standard deviation (SD). If the continuous variables can meet a normal distribution and homoscedasticity, One way analysis of variance (ANOVA) with multiple comparisons will be applied to identify differences more than two groups, while Student *t* test for pairwise comparison. Otherwise, we will use Tamhane T2 method instead. Halberg cosiner software will be used to analysis the circadian rhythm. *P* value is under .05 will be taken as statistically significant for each outcome. We will select the intention-to-treat principle to perform this statistical analysis. Thus, missing data will be imputed by using the multiple imputation method.

We will use two different methods: intention-to treat and per-protocol, for sensitivity analysis.

Halberg cosiner software will be utilized to analysis the circadian rhythm. For using the program, we will create time series per file (with the time tag, mean and SD). Then, the program will fit the cosine curve and polar coordinates and judge whether the parameters can show a circadian rhythm. By the formula of cosine curve, the results will be given, such as mesor, amplitude, acrophase. The results were considered significant when the *P* value <.05.

### 2.14. Safety and AEs

The group A and B may encounter AEs including allergic reactions, skin burn, general fatigue, stomach upsets, headaches, bleeding tendency, dizziness, fainting and constipation.^[17]^ If the above condition occurs, the Du moxibustion treatment should be stopped immediately. These AEs will be subcategorized by severity: mild, moderate, and severe AEs (mild AEs = AEs are transient and tolerable; moderate AEs = AEs will cause discomfort and interfere with the subject’s normal life; severe AEs = serious impact on the participants’ physical health and even lead to the risk of life). The record form will be filled if AEs occur during the treatment period including the time, duration, performance, measures to be taken, and the outcome.

### 2.15. Quality control

A standardized case report form with detailed record rules will be used in order to ensure accurate data collection in the clinical trial. Additionally, the research assistants will regularly communicate with participants every day through WeChat, telephone or short message to improve patient compliance. Each staff will be required to receive corresponding training according to their respective responsibilities in this trial, including patients recruitment, randomization, instructing patients filling out the forms, moxibustion intervention, effect assessment and data management before the research. The moxibustion operators need to have gained medical licenses in China besides accepting strict training. All investigators are qualified to conduct this study after they pass the examination. Furthermore, to ensure the quality of this trial, a clinical proctor will check the processes of the trial and document the details in the hospital at regular intervals. In addition, double data entry will be applied and monitors who are nominated by the principal investigator will verify the accuracy and validity of the original data. Finally, regular meetings will be held to discuss any practical issues appearing during this study and propose solutions, as well as report the AEs, protocol violation, recruitment rate. At the end of the study, the cause of patient drop-out should be recorded in the case report form for all shedding cases as much as possible.

### 2.16. Patients and public involvement

Participants or patients in this trial will not be involved in the design, or conduct, or reporting, or dissemination plans of the study.

### 2.17. Ethics

This study protocol will be carried out in accordance with the Declaration of Helsinki, and it was approved by the Medical Ethics Committee Board of the Affiliated TCM Hospital of Southwest Medical University, Luzhou, Sichuan, China (number: KY2021065-FS01). Once recruited, each participants would be requested to voluntarily sign a written informed consent form as acceptance in this research study before randomization. The present study is financially supported by the funds for the special project of scientific research on TCM Bureau of Sichuan Province (No. 2021MS084) and the National Natural Science Foundation of China (NO. 81804208). This study protocol has been registered at Chinese Clinical Trials Registry with the identifier number: ChiCTR2100051532.

## 3. Discussion

The main pathological mechanism of RA is synovitis and the main feature of its clinical manifestations possess circadian variation which shows light symptoms during the day and heavy symptoms at night.^[18]^ Basic research discovered that the rhythm variations of clinical feature were attributed to RA pathological rhythm, which referred to the rhythmic oscillation of inflammatory cytokines Additionally, the achievements of chronotherapy in Western medicine have prompted us to associate RA pathology with chronotherapy in TCM. Moxibustion is an important part of TCM and can also provide chronotherapy which is called Chrono-moxibustion. Previous research found that moxibustion is effective in RA treatment and can inhibit the inflammatory response of RA by regulating pro-inflammatory cytokines.^[10]^ Besides, Du moxibustion can warm Yang and has definite curative effect on RA. Consequently, we will assess the effectiveness of Chrono-moxibustion and explore its influence on circadian rhythm of RA patients.

TCM believes that the external cause of the RA disease is the feeling of wind, cold and dampness evil, while the basic cause is the deficiency of vital qi, which means the syndrome differentiation is Yang deficiency. The general treatment strategies of RA are drug therapies such as NSAIDs, DMARDs, and Glucocorticoids in western medicine, which may result in serious side-effects on various systems of body if used for a long time.^[4]^ Nevertheless, TCM has a proven remarkable effect on RA as supplemental treatment such as acupuncture, massage, and moxibustion.

Previous studies found that moxibustion treatment can relieve morning stiffness, joint swelling, pain and other clinical symptoms of RA patients and improve their quality of life.^[9]^ Moxibustion could not only increase the effect on clinical symptoms, but also decrease the serum level of IL-1, TNF-α, MMP-1, and MMP-3, which were serological markers related to bone and cartilage metabolism of RA.^[11]^ Therefore, we decided to adopt moxibustion as an intervention in this study.

Du moxibustion is mainly applied to the Du Meridian of the human body, which is the sea of Yang vessels and can regulate the Qi and blood of the whole body and modify the activity of Yang Qi. Therefore, Du moxibustion can warm and regulate the Yang Qi of meridians and collaterals, and help Yang to dispel pathogenic factors like wind, cold and dampness. Compared with ordinary moxibustion, Du moxibustion has the advantages of large coverage, powerful firepower, high safety, simple operation, low price, easy control and low scald risk, which is a simple and effective treatment for RA patients.

Previous findings also suggested that IL-6 levels in RA patients showed significant circadian rhythm changes that peak values appeared in the morning and low values in the evening, which were consistent with the diurnal change of symptoms. Further, some scholars found that moxibustion might play an important anti-inflammatory role by modulating the circadian oscillation of IL-6 and TNF-α in rats.^[19,20]^ Nonetheless, the evaluation of the clinical efficacy of moxibustion in RA treatment is relatively limited. For now, there is a lack of human research in terms of regulating circadian rhythm. Therefore, we hypothesized that moxibustion can ameliorate the symptoms of RA by improving circadian rhythm. In this trial, except the questionnaires to estimate the clinical efficacy, we also have many laboratory indicators, as well as Halberg cosiner software will be utilized which will reveal the circadian rhythm mechanism of moxibustion in RA treatment and provide clear, effective, and intuitive data.

Correlation between RA and circadian rhythm have been reported in literatures. Previous experiments found that the pathological rhythm of RA was closely related to the circadian oscillation of inflammatory factors such as IL-1β, IL-6, and TNF-α.^[19,20]^ The circadian clock system in humans consists of a central clock and peripheral clocks. The central clock is located in the suprachiasmatic nucleus of the hypothalamus, while the peripheral clocks widely exists in other tissues and organs of whole body. Circadian rhythm is regulated by the endogenous circadian clock genes which were involved in RA pathology. RA inflammation can disrupt the expression of clock gene, leading to further aggravation of synovitis. Similarly, due to the crosstalk between circadian clock and inflammation, disorders of circadian clock can aggravate inflammation. Experiment has shown that the expression of BMAL1 mRNA in LPS induced arthritis mice was lower than that in control group, and it pointed out that BMAL1 play an vital role in anti-inflammation when monocytes and macrophages were attacked by immune stimulants.^[21]^ Another study discovered that after serum shock, the expression level of BMAL1 mRNA in RA synovial fibroblasts was much lower than that in OA control group, and the peak expression was delayed.^[22]^

Thus, we designed this trial to evaluate the effectiveness of Chrono-moxibustion in RA treatment, as well as to explore its influence on diurnal rhythm of RA patients. Nevertheless, there are also limitations to this study. First of all, the blinding is one of the limitations. Due to the nature of moxibustion, blinding of participants and personnel was not possible, which may lead to high risk of performance bias. For this reason, we will strictly conduct randomization, while assessors and statisticians will be blinded to adjust bias. Besides, patients in group A and B may get more chance to contact with their moxibustion operators and construct a good therapist-patient interaction rather than those in group C, which is more likely to enhance the therapeutic effect.^[23]^ To minimize this potential bias as much as possible, we will keep close contact with patients in group C by telephone, emails, Internet, and so on. Finally, it is important to note that the participants will only be recruited in China, so there may be insufficient representation.

In summary, the results of this research will be expected to confirm whether Chrono-moxibustion can alleviate RA, and point out which time the effect will be better, so as to help doctors and patients to select an optimal treatment time. Combined with the laboratory indicators results, it will help us to reveal the mechanism of Moxibustion in the treatment of RA. We believe that the results will engender a positive impact on moxibustion in the treatment of RA.

## Author contributions

**Conceptualization:** Mingfang Yu, Shenqiao Yang, Xiao Wu.

**Data curation:** Xinling He, Aiyang Wang.

**Methodology:** Mingfang Yu, Shenqiao Yang.

**Project administration:** Bailu Chen, Lu Gan.

**Writing – original draft:** Mingfang Yu, Shenqiao Yang.

**Writing – review & editing:** Mingfang Yu, Shenqiao Yang, Xiao Wu.
